# “Decolonising” the University of Edinburgh Medical School Psychiatry Curriculum

**DOI:** 10.1192/bjo.2022.142

**Published:** 2022-06-20

**Authors:** Emily Nelson, Deborah Cooper, Pujit Gandhi, Heather King, Elizabeth Lloyd, Jamie Burrell, Anushka Pathak

**Affiliations:** 1 NHS Lothian, Edinburgh, United Kingdom; 2 NHS Fife, Cupar, United Kingdom.; 3 University of Edinburgh, Edinburgh, United Kingdom

## Abstract

**Aims:**

The concept of “decolonisation” has gradually evolved within higher education, and can be defined as seeking to discern how historical systems of discrimination have shaped the networks around us, and how to adjust to the perspectives of those who have been oppressed and minoritised by these systems. Our aim was to assess what gaps there are in the Edinburgh Medical School psychiatry curriculum, in order that this might inform our next steps in “decolonising” the curriculum.

**Methods:**

We reviewed all the teaching materials used for teaching Year 5 Psychiatry at the University of Edinburgh (n = 101). We made a count of the number of people or cases in each resource and the diversity of examples used. We subsequently examined each resource to see if it touched on each of six key areas considered to be representative of a “decolonising” effort. These were the assignment of gender only where necessary, cultural/religious differences, historical context, health inequalities, the patient-doctor relationship and global topics.

**Results:**

Of the resources where each of the criteria were applicable, 18% only assigned gender where necessary or left gender neutral, 4.35% addressed cultural or religious differences, 5.8% discussed the historical context, 4.35% tackled health inequalities, 1.45% raised the doctor-patient relationship and none introduced global topics. Of all the resources that include a direct reference to a patient or case, only 5.41% were explicitly from a different ethnic group other than “white”.

**Conclusion:**

Our results show that all the key areas can be improved on. Addressing these issues has not been a focus for the curriculum before now and our next steps will be to approach each topic in turn and consider how the key areas can be introduced. We are assembling a focus group of psychiatrists and medical students and have designed a survey for students who have completed their psychiatry block.

With time, we hope to cultivate an attitude amongst students and teachers of psychiatry at Edinburgh University that boldly confronts the historical development of our subject, acknowledges those who have suffered for it, picks up on what may be missing or misrepresented, and encourages critical analysis of research. Our teaching materials should include examples which explore stereotypes and challenge prejudices. By broadening our repertoire, confronting the darker parts of our history, listening to those with quieter voices, and paying attention to lived experience, we can foster a culture of teaching and learning which is open, flexible and humble.

